# An Emerging Translational Model to Screen Potential Medicinal Plants for Nephrolithiasis, an Independent Risk Factor for Chronic Kidney Disease

**DOI:** 10.1155/2014/972958

**Published:** 2014-07-06

**Authors:** San-Yuan Wu, Jui-Lung Shen, Kee-Ming Man, Yuan-Ju Lee, Huey-Yi Chen, Yung-Hsiang Chen, Kao-Sung Tsai, Fuu-Jen Tsai, Wei-Yong Lin, Wen-Chi Chen

**Affiliations:** ^1^School of Chinese Medicine, Graduate Institute of Chinese Medicine, Graduate Institute of Integrated Medicine, College of Chinese Medicine, Research Center for Chinese Medicine and Acupuncture, China Medical University, Taichung 40402, Taiwan; ^2^Center for General Education, Feng Chia University, Taichung 40724, Taiwan; ^3^Department of Dermatology, Taichung Veterans General Hospital, Taichung 40705, Taiwan; ^4^Department of Medicinal Botanicals and Health Applications, Da-Yeh University, Changhua 51591, Taiwan; ^5^Department of Anesthesiology, Tungs' Taichung Harbor Hospital, Taichung 43304, Taiwan; ^6^Department of Life Sciences, National Chung Hsing University, Taichung 40227, Taiwan; ^7^Graduate Institute of Geriatric Medicine, Anhui Medical University, Hefei 230032, China; ^8^Department of Urology, National Taiwan University Hospital, Taipei 10002, Taiwan; ^9^Departments of Medical Research, Obstetrics and Gynecology, Dermatology, and Urology, China Medical University Hospital, Taichung 40447, Taiwan

## Abstract

Pharmacological therapy for urolithiasis using medicinal plants has been increasingly adopted for the prevention of its recurrence. A *Drosophila melanogaster* model developed for translational research of urolithiasis was applied to evaluate agents with potential antilithic effects and calcium oxalate (CaOx) formation. Potential antilithic herbs were prepared in a mixture of food in a diluted concentration of 5,000 from the original extract with 0.5% ethylene glycol (EG) as the lithogenic agent. The control group was fed with food only. After 3 weeks, flies (*n* ≥ 150 for each group) were killed using CO_2_ narcotization, and the Malpighian tubules were dissected, removed, and processed for polarized light microscopy examination of the crystals. The crystal formation rate in the EG group was 100.0%. In the study, 16 tested herbal drugs reached the crystal formation rate of 0.0%, including *Salviae miltiorrhizae*, *Paeonia lactiflora*, and *Carthami flos*. *Scutellaria baicalensis* enhanced CaOx crystal formation. Two herbal drugs *Commiphora molmol* and *Natrii sulfas* caused the death of all flies. Our rapid screening methods provided evidence that some medicinal plants have potential antilithic effects. These useful medicinal plants can be further studied using other animal or human models to verify their effects.

## 1. Introduction

Urolithiasis is a common urologic disorder with high prevalence and recurrence [1–4]. It has been reported that kidney stones are a significant and independent risk factor for chronic kidney disease in the general population. An overall prevalence from 9.6% to 6.5% and a 5-year recurrence rate of 34.7% are observed in Taiwan [5, 6]. Owing to high recurrence, various treatment modalities are available; however, currently, the prevention of the recurrence of urolithiasis remains a challenge [7].

Pharmacological therapy for urolithiasis using medicinal plants in traditional Chinese medicine (TCM) has been arising for the prevention of its recurrence [8–12]. A single agent or formula such as Takusha, Wulingsan, and* Desmodium styracifolium* were reviewed by Miyaoka and Monga. They concluded that TCM has promising roles in urinary stone prevention [13]. However, over 300 different types of medicinal plants exist according to the record of “Ben Cao Bei Yao (*本草備要*; Complete Essentials of the Materia Medica),” a famous TCM pharmacology book. Few of them were studied for the antilithic effect, although potential effects were recorded. One of the possible causes for this is the lack of rapid tools to extensively study these abundant herbal drugs [14–16].

The development of novel translational research technologies and approaches is of central importance for successful complementary and alternative (CAM) research. Recently, we have developed a fruit fly (*Drosophila melanogaster*) model for translational research of urolithiasis and applied this model to the evaluation of agents that may have potential antilithic effect [17–19]. The Malpighian tubule of the fruit fly has a function similar to the human kidney [20–23].

Calcium oxalate (CaOx) crystal formation can be seen 3 weeks after the addition of lithogenic agents (ethylene glycol (EG)) to the food. The crystals were easily observed under polarized microscopy, and the crystal formation rate can then be evaluated [17–19]. For example, a test for the effect of melamine on crystal formation in* Drosophila* was performed. The results indicate that the administration of melamine caused crystal formation in a mixture of CaOx, calcium phosphate, uric acid, and melamine crystals [18]. In a previous study by Ho et al., it was found that potassium citrate could prevent crystal formation in EG-induced CaOx nephrolithiasis in* Drosophila.* No inhibitory capability of commercial citrate-containing juice was observed for CaOx crystal formation in the Malpighian tubules of the fly [19]. Therefore, this model can be further used as a rapid screening method to test any drugs that have a potential antilithic effect. Since kidney stones are a significant and independent risk factor for chronic kidney disease in the general population. In this study, we attempted to use this emerging translational model to rapidly screen the potential antilithic medicinal plants.

## 2. Materials and Methods

### 2.1. Animal Model

We used wild-type male* D. melanogaster* fed with 0.5% EG as lithogenic group and* D. melanogaster* fed without lithogenic agent as control group in this study for the evaluation of CaOx crystal formation [17–19]. In brief, flies were bred in plastic vials containing a medium of yeast, corn syrup, and agar. Flies were maintained under a condition of 25°C, 60% humidity, and a 12 h light-dark cycle. Potential antilithic herbs were prepared in a mixture of food in a diluted concentration of 5,000 from the original extract with 0.5% EG (Sigma, USA) as the lithogenic agent. The control group of flies was fed with food only. After 3 weeks, flies (*n* ≥ 150 for each group) were killed by CO_2_ narcotization, and the Malpighian tubules were dissected, removed, and processed for examination of the crystals by polarized light microscopy.

### 2.2. Screening of Potential Antilithic Medicinal Plants

The extracts of herbs were provided by Sun Ten Pharmaceutical Co. (Taichung, Taiwan). We then selected potential herbs according to the record of “Ben Cao Bei Yao.” A total of 80 herbs were tested in this study (Table 1). Tested herbal drugs were considered to have an antilithic effect if the crystal formation rate was zero. The total death of flies in the tested group was not considered for the positive effect.

### 2.3. Observation of CaOx Crystal Formation

The Malpighian tubules were dissected and immediately observed under normal and polarized white light using an Olympus BX51 optical microscope (Japan) after crystal induction. We photographed the relevant crystal aspects using a Kodak ProImage 100 film with scales (USA).

### 2.4. Statistical Analysis

For each group, data are presented as the crystal formation rate (%). All calculations were performed using Statistical Package for Social Sciences (SPSS for Windows, version 8.0, SPSS Inc., Chicago, IL, USA).

## 3. Results

### 3.1. CaOx Crystal Formation Rate

**Figure 1:**
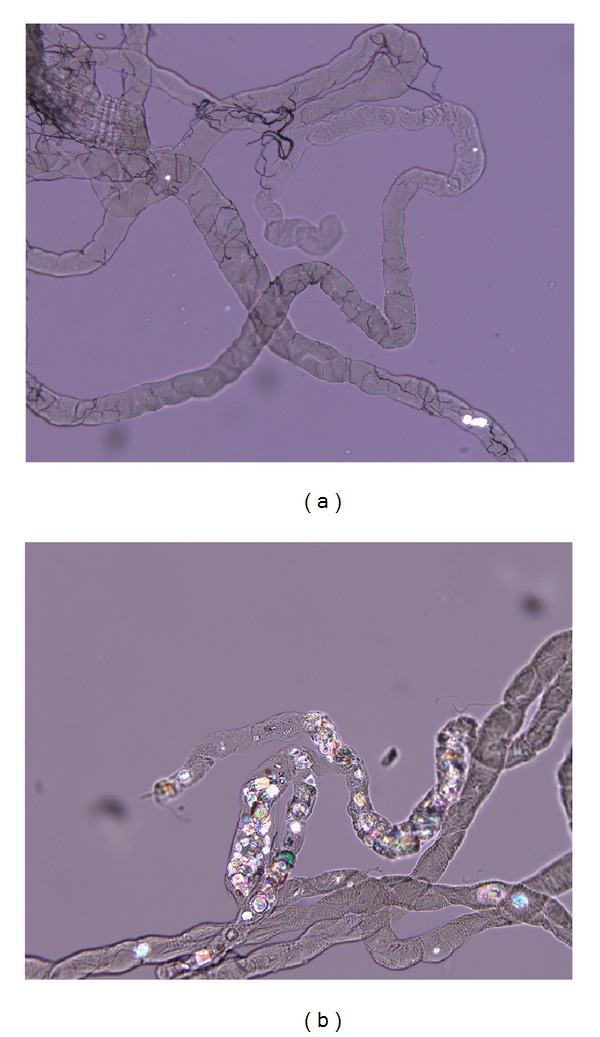
EG-induced CaOx crystal deposition in the Malpighian tubules. The images show representative polarized microscopy for the (a) control flies and (b) the flies with 0.5% EG-induced crystal formation in Malpighian tubules.

The crystal formation rate in EG and control groups was 100.0% and 10.2%, respectively. Positive CaOx crystal formation can be seen in the Malpighian tubules of flies (Figure 1). In the first study, 16 tested herbal drugs reached the crystal formation rate of 0% (Table 1), namely,* Salviae miltiorrhizae* (number 11, *丹參*),* Paeonia lactiflora* (number 19, *白芍藥*),* Carthami flos* (number 21, *紅花*),* Corydalis yanhusuo* (number 29, *延胡索*),* Imperata cylindrica* (number 35, *白茅根*),* Prunus armeniaca* (number 42, *杏仁*),* Eclipta prostrata* (number 43, *旱蓮草*),* Artemisia argyi* (number 46, *艾葉*),* Plantago asiatica* (number 48 and its seed number 50, *車前子*),* Lonicera japonica* (number 49, *忍冬藤*),* Polygoni cuspidati* (number 52, *虎杖*),* Astragalus membranaceus* (number 67, *黃耆*),* Wolfiporia cocos *(number 70, *茯苓*),* Scutellaria baicalensis *(number 76, *黃芩*), and* Angelicae sinensis* (number 77, *當歸*).

Two herbal drugs caused the death of all flies in this study. These were* Commiphora molmol* (number 44, *沒藥*) and* Natrii sulfas* (number 47, *芒硝*), and death may be due to their toxicities.

In contrast,* Cuscuta chinensis* (number 78, *菟絲子*) enhanced CaOx crystal formation in the Malpighian tubules, reaching the crystal formation rate of 100%.

## 4. Discussion

In our survey, 16 herbs were determined to be successful antilithic herbs. Our results provide valuable information for future studies regarding antilithic herbs suitable for the prevention of urolithiasis. In an ongoing study,* Carthami flos* has been further studied for its potential antilithic effects, and a positive result was obtained when it was applied to a rat model [11].

Abundant herbal drugs with antilithic effects that are used for treatment are available in TCM books [17, 24–27]. Some drugs have been studied using in vitro models such as nucleation, crystal aggregation, and crystal growth [23]. Conventionally, rats have been used as animal models to study the crystal formation rate in the kidney. The application of a large number of herbal drugs requires the use of a large number of animals, leading to high cost. Our study used a large number of animals and large-scale drug lists to reach significant results that provide valuable data for the study of antilithic effects. The use of the fruit fly has been cited as a potential new animal model to study urolithiasis and has been proven to be effective [20, 23].

*Cuscuta chinensis* has several pharmacological effects, particularly in the genitourinary tract, including replenishing the kidney essence and improving sperm motility, kidney deficiency, urinary frequency, and erectile dysfunction [28, 29]. Its effectiveness was reported by Pan et al.; they showed that an ethanol extract of* Cuscuta chinensis* was effective on Th1 and Th2 cell functions and that it could be safely used as an adjuvant in a mice model [30]. A patent (publication number CN102579706 A; 2012) has been used for the application of* Cuscuta chinensis* as part of a formula for treating urolithiasis in China. This formula reached an effective rate of 70%. We also selected* Cuscuta chinensis* as a potential antilithic agent in this study. However, the treatment detail of this formula remains unclear because this formula is not composed of a single herb. Based on our results,* Cuscuta chinensis* exerted adverse effects that completely enhanced crystal formation. Therefore, further studies will need to be performed to clearly identify the effects of individual components by other means.

*Natrii sulfas* is a TCM drug made from crystals refined from the processed Glauber's salt, which is crystalline hydrated form of sodium sulfate. It mainly contains Na_2_SO_4_ *·*10H_2_O. In a study on rats,* Natrii sulfas* was suggested to have a protective effect on ischemia-induced brain edema and to improve the physiological symptoms [31]. It has been recorded in the TCM books that* Natrii sulfas* can cause the lysis of urinary stones, but it caused the death of flies in this study. Although these herbal drugs may have potential antilithic effects, they have been revealed to have toxic effects; therefore, they cannot be used as a single agent for the treatment of urolithiasis. Conventionally, TCM uses a formula with a combination of several drugs to reduce the toxic effect. These drugs should be carefully reconsidered if used clinically.

*Myrrha* is a resin made from the* Commiphora molmol* tree. Studies on* Myrrha* revealed analgesic and anti-inflammatory effects in the treatment of various diseases associated with inflammatory pain, such as arthritis, obesity, microbial infection, wound, pain, fractures, tumor, and gastrointestinal diseases [32, 33]. In this study, we selected* Myrrha* because it is used for spasm and pain relief. However, in our study,* Myrrha* caused the death of flies. Some side effects have been associated with* Myrrha*, such as bleeding tendency, damage to the kidney, or stomach pain [34, 35]. The use of smaller dosage as part of the herbal formula has been suggested. The limitation of this herb is that it caused the death of flies. Further studies using smaller dosage may elucidate the effect of* Myrrha* on the treatment of urolithiasis.

An advantage of our study is that a large number of types of herbs could be rapidly screened within a short period using a large number of animals. The limitations of this study include crude water extracts only, limited dosage control, and invertebrate animal model, which may have important anatomical differences compared with mammals. However, this model has proven to be reliable, since several scientific research articles have been published with this stable fly model.

## 5. Conclusion

The development of novel translational research technologies and approaches is of central importance for successful CAM research [36–39]. For long, a huge disconnect has been observed between clinical and preclinical studies of CAM agents. Our rapid screening methods provide evidence that some traditional herbs have a potential antilithic effect. These useful crude extracts can be further studied using other animal or human models to verify their effects, which have been recorded in TCM books.

## Figures and Tables

**Table 1:** Potential antilithic medicinal plants/agents and rate of calcium oxalate crystal formation in Malpighian tubules of male *Drosophila*.

Common name	Latin name/chemical name	Crystal formation (%)
Control		10.20
0.5% EG		100.00
Chinese hawthorn	*Crataegus pinnatifida *	36.36
Dogwood	*Cornus officinalis *	62.50
Chinese yam	*Dioscorea opposite *	45.45
Sichuan lovage rhizome	*Ligusticum chuanxiong *	27.27
Pseudoginseng	*Panax notoginseng *	33.33
Common bur reed	*Sparganium stoloniferum *	45.45
Szechwan chinaberry	*Melia toosendan *	30.00
Rhubarb	*Rheum rhabarbarum *	25.00
Morinda root	*Morindae officinalis. *	33.33
Trogopterus dung	*Faeces trogopterori *	7.14
Salvia root	*Salviae miltiorrhizae *	0.00
Cowherb seed	*Semen vaccariae *	20.00
Achyranthes root	*Achyranthis bidentatae *	30.00
Costus root	*Aucklandia lappa *	20.00
Kidney tea	*Clerodendranthus spicatus *	30.00
*Akebia* caulis	*Caulis akebiae *	10.00
Licorice root	*Glycyrrhiza uralensis *	40.00
Largehead atractylodes	*Atractylodis macrocephalae *	80.00
White peony root	*Paeonia lactiflora *	0.00
Dried rehmannia root	*Rehmannia glutinosa *	30.00
Safflower	*Carthami flos *	0.00
Dark plum fruit	*Fructus mume *	60.00
Bupleurum	*Bupleurum chinensis *	54.55
Magnolia bark	*Magnolia officinalis *	50.00
Peach kernel	*Semen persicae *	25.00
Immature bitter orange	*Citrus aurantium *	50.00
Fructus aurantii	*Citrus aurantium Fructus *	40.00
Wolfberry	*Lycium barbarum *	66.67
Corydalis tuber	*Corydalis yanhusuo *	0.00
Walnut	*Juglans regia *	30.00
Herba lysimachiae	*Lysimachia christinae *	9.00
Common monkshood root	*Aconitum carmichaelii *	40.00
Chinese clematis	*Clematis chinensis *	28.57
Fructus evodiae	*Evodia rutaecarpa *	18.18
Lalang grass rhizome	*Imperata cylindrica *	0.00
Cinnamon	*Cinnamomum cassia *	8.33
Ginger	*Zingiber officinale *	10.00
Chingma abutilon seed	*Abutilon indicum *	41.67
Taiwan angelica root	*Angelica dahurica *	25.00
Cynanchum glaucescens	*Cynanchi stauntonii *	8.33
Blackened swallowwort root	*Cynanchum atratum *	9.09
Almond	*Prunus armeniaca *	0.00
Yerbadetajo herb	*Eclipta prostrata *	0.00
Myrrh	*Commiphora molmol *	—
Common peony root	*Paeonia veitchii *	64.00
Argy wormwood leaf	*Artemisia argyi *	0.00
Glauber's salt	Sodium sulfate	—
Plantaginis seed	*Plantago asiatica* L.	0.00
Honeysuckle stem	*Lonicera japonica *	0.00
Plantaginis	*Plantago asiatica *	0.00
Frankincense	*Boswellia sacra *	11.00
Giant knotweed	*Polygoni cuspidati *	0.00
Honeysuckle flower	*Lonicera japonica* Thunb.	18.00
Polyporus	*Polyporus umbellatus *	75.00
Talcum powder	Pulvis talci	40.00
Taraxacum	*Taraxacum officinale *	57.14
Membrane of chicken gizzard	N/A	66.67
Common rush	*Juncus effusus *	28.57
Carapax trionycis	*Trionyx sinensis *	57.14
Rehmanniae preparata root	*Rehmannia glutinosa *	44.44
Bazheng powder	N/A	33.00
Fringed pink	*Dianthus superbus *	75.00
Water plantain	*Alisma canaliculatum *	25.00
Coix seed	*Coix lacryma-jobi *	75.00
Pilose asiabell root	*Codonopsis pilosula *	33.33
Himalayan teasel root	*Dipsacus asperoides *	100.00
Milkvetch root	*Astragalus membranaceus *	0.00
Cape jasmine fruit	*Gardenia jasminoides *	50.00
Rhizoma curcumae	*Curcuma phaeocaulis *	50.00
Indian buead	*Wolfiporia cocos *	0.00
Sweetgum fruit	*Liquidambar formosana *	20.00
Rice paperiant pith	*Tetrapanax papyriferus *	100.00
Combined spicebush root	*Lindera aggregata *	40.00
Corn stigma	*Zea mays *L.	66.00
Common knotgrass	*Polygonum aviculare *L.	50.00
Baical skullcap root	*Scutellaria baicalensis *	0.00
Angelica root	*Angelicae sinensis *	0.00
South dodder seed	*Cuscuta chinensis *	100.00
Scurfpea fruit	*Psoralea corylifolia *	25.00
Dwarf lilyturf tuber	*Ophiopogonjaponica *	50.00
