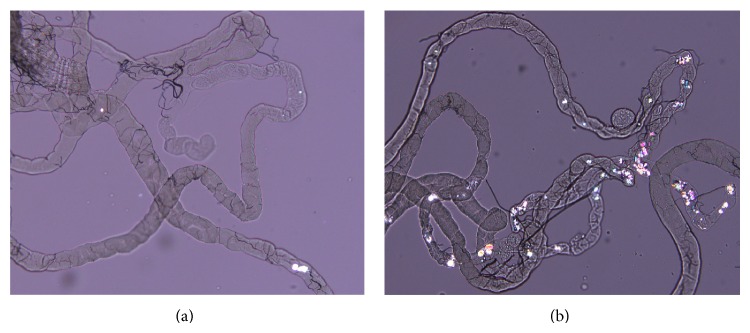# Corrigendum to “An Emerging Translational Model to Screen Potential Medicinal Plants for Nephrolithiasis, an Independent Risk Factor for Chronic Kidney Disease”

**DOI:** 10.1155/2015/713703

**Published:** 2015-02-23

**Authors:** San-Yuan Wu, Jui-Lung Shen, Kee-Ming Man, Yuan-Ju Lee, Huey-Yi Chen, Yung-Hsiang Chen, Kao-Sung Tsai, Fuu-Jen Tsai, Wei-Yong Lin, Wen-Chi Chen

**Affiliations:** ^1^School of Chinese Medicine, Graduate Institute of Chinese Medicine, Graduate Institute of Integrated Medicine, College of Chinese Medicine, Research Center for Chinese Medicine and Acupuncture, China Medical University, Taichung 40402, Taiwan; ^2^Center for General Education, Feng Chia University, Taichung 40724, Taiwan; ^3^Department of Dermatology, Taichung Veterans General Hospital, Taichung 40705, Taiwan; ^4^Department of Medicinal Botanicals and Health Applications, Da-Yeh University, Changhua 51591, Taiwan; ^5^Department of Anesthesiology, Tungs' Taichung Harbor Hospital, Taichung 43304, Taiwan; ^6^Department of Life Sciences, National Chung Hsing University, Taichung 40227, Taiwan; ^7^Graduate Institute of Geriatric Medicine, Anhui Medical University, Hefei 230032, China; ^8^Department of Urology, National Taiwan University Hospital, Taipei 10002, Taiwan; ^9^Departments of Medical Research, Obstetrics and Gynecology, Dermatology, and Urology, China Medical University Hospital, Taichung 40447, Taiwan

We have noticed an inadvertent error in our paper “An Emerging Translational Model to Screen Potential Medicinal Plants for Nephrolithiasis, an Independent Risk Factor for Chronic Kidney Disease” [1].

There is an error that occurred during uploading Figure 1(b). The published representative polarized microscopy image for the flies with 0.5% EG-induced crystal formation in Malpighian tubules is incorrect. We have attached a corrected version of Figure 1. This error does not change the scientific conclusions of the paper in any way.

## Figures and Tables

**Figure 1:**